# Editorial: Sparkling wines: current trends and future evolution

**DOI:** 10.3389/fmicb.2023.1199578

**Published:** 2023-05-10

**Authors:** Giorgia Perpetuini, Rosanna Tofalo

**Affiliations:** Department of Bioscience and Technology for Food, Agriculture and Environment, University of Teramo, Teramo, Italy

**Keywords:** sparkling wine, yeast, autolysis, fermentation, Maillard reaction

Sparkling wines belong to the category of special wines and are “characterized on uncorking by the production of a more or less persistent effervescence resulting from the release of CO_2_ (the excess pressure of this gas in the bottle is at least 3.5 bars at 20°C) of exclusively endogenous origin” (OIV Focus, 2023).

Sparkling wine production is based on two fermentation phases: in the first, the base wine is produced through the alcoholic fermentation process; (ii) in the second, the base wine is supplemented with sucrose, selected yeasts, and other additives in order to start the fermentation process. This step can take place in the bottle (traditional method) or in an autoclave (Martinotti-Charmat method). An aging period in the cellar follows this phase, during which yeast autolysis occurs and different metabolites are released, enriching the aroma character of the sparkling wine (Tofalo et al., 2022).

Over the last few decades, the international wine market has dramatically changed and sparkling wine production is on the rise and shows no sign of slowing down. A general tendency is to diversify sparkling wines. To achieve this aim, the potentiality of different yeast strains is under study to diversify sparkling wines and to associate them to a specific wine producing area.

A possible strategy is the exploitation of non-*Saccharomyces* yeasts. Among non-*Saccharomyces* yeasts, *Torulaspora delbrueckii* has been proposed as starter culture. Unfortunately, this yeast is less effective than *Saccharomyces cerevisiae* at completing wine fermentation.

Martinez et al. performed the hybridization of spores and vegetative cells of a *T. delbrueckii* killer strain with a *S. cerevisiae* cycloheximide resistant strain in order to ensure the transfer of genetic information from *S. cerevisiae* to *T. delbrueckii*. The obtained hybrids showed improved fermentation kinetics, cell survival, and foam quality. Additionally, yeast mixture clones are stable enough to be marketed for industrial sparkling winemaking.

An important aspect of sparkling and still wine production is their aroma profile. The synthesis of aroma compounds depends on several factors: grape cultivar, must composition, the yeast strain, fermentation temperature, and nitrogen, which is a limiting nutrient for yeasts' development during the fermentation process. Organic and inorganic nitrogen is naturally present in the grape must, but extra nitrogen can be added to overcome the problem of slow fermentations. Godillot et al. investigated the impact of nitrogen addition during the stationary phase on yeast metabolism and especially on fermentative aromas synthesis. The production of amino acids and the central carbon metabolism were shown to be triggered by the addition of nitrogen. It was also demonstrated that adding nitrogen at the start of the stationary phase increased the conversion of higher alcohols into acetate esters. They also showed that *ATF*1 gene expression was constitutive and not susceptible to nitrogen increases, whereas *ATF*2 gene expression was inducible. Thus, rather than the availability of their respective precursors, it seems that, the crucial component in the synthesis of both acetate and ethyl esters is the enzymatic activity responsible for their formation. However, the regulation of ester formation is different as a result of nitrogen addition during the stationary phase: for acetate esters, it is related to the degree of gene *ATF*2 expression, but for ethyl esters, it appears to be allosterically regulated.

Research on the formation of Maillard associated products in sparkling wine may reveal strategies to achieve enhanced aroma in the production of traditional method sparkling wines and identify links to the production process. Charnock et al. presented a comprehensive review about the formation of Maillard reaction-associated compounds in sparkling wines. Maillard reaction pathways in traditional sparkling wine are discussed. Moreover, the factors that may contribute to the formation of associated compounds are evaluated with a focus on the composition and origins of precursor species during the production process.

The fruit wine market is currently moving toward diversification through the development of innovative formulas, technologies, and alternative raw materials. Among fruit wines, tangerine wine is a type of fruit wine produced in the Nanfeng district (Jiangxi province – China), and it is characterized by a low alcohol concentration. The fermentation process is complex and several microbial populations are involved. Qiu et al. determined the structure and function of core microorganisms involved in the fermentation process of tangerine wine during spontaneous and inoculated fermentation with a commercial strain of *S. cerevisiae*. *Lactobacillus* and *Acetobacter* were the main genera detected in both fermentations, while *Hanseniaspora, Pichia*, and *Saccharomyces* were the main fungal genera. The correlation analysis between microbiota and aroma compounds revealed that *Lactobacillus, Acetobacter, Hanseniaspora*, and *Saccharomyces* were the main genera contributing to the production of the volatile profile of Nanfeng tangerine wine.

Similarly, Zhao et al. collected 92 samples of rice wine koji from agricultural produce markets in the Hubei, Sichuan, and Guangxi provinces of China. They investigated the rice wine koji microbiota and its contribution to the fermentation process. Firmicutes and Proteobacteria were the dominant phyla; however, the bacterial community structure of rice wine koji samples from different regions was significantly different. The authors suggested that the differences detected could be related to the environment, or manufacturing. Therefore, the wine koji could be a source of new bacterial strains to enhance the industrial production of rice wine.

The exploitation of microbial biodiversity could improve the fermentation process of sparkling and still wines. However, additional research is still required to exploit the enormous potential associated with natural microbial biodiversity. Moreover, the adoption of genetic approaches to characterize emerging microbial consortiums could supply new tools to improve sparkling wine production.

## Author contributions

GP and RT contributed to the conception, design of the Research Topic, and wrote the editorial. Both authors contributed to manuscript revision, read, and approved the submitted version.

## References

[B1] OIV Focus—The Global Sparkling Wine Market. (2023). Available online at: https://www.oiv.int/public/medias/7291/oiv-sparkling-focus-2020.pdf (accessed March 24, 2023).

[B2] Tofalo R Perpetuini G Rossetti A. P Gaggiotti S Piva A Olivastri L. . (2022). Impact of *Saccharomyces cerevisiae* and non-*Saccharomyces* yeasts to improve traditional sparkling wines production. Food Microbiol. 108, 104097. 10.1016/j.fm.2022.104097 36088113

